# Association of cognitive performance with clinical staging in schizophrenia spectrum disorders: a prospective 6-year follow-up study

**DOI:** 10.1016/j.scog.2021.100232

**Published:** 2021-12-14

**Authors:** S. Berendsen, E. Nummenin, F. Schirmbeck, L. de Haan, M.J. van Tricht, GROUP Investigators, Amelsvoort, Agna A. Bartels-Velthuis, Lieuwe de Haan, Frederike Schirmbeck, Claudia J.P. Simons

**Affiliations:** 1 University of Groningen, University Medical Center Groningen, University Center for Psychiatry, Rob Giel Research Center, Groningen, the Netherlands; 2 Amsterdam UMC, University of Amsterdam, Department of Psychiatry, Amsterdam, the Netherlands; 3 Maastricht University Medical Center, Department of Psychiatry and Neuropsychology, School for Mental Health and Neuroscience, Maastricht, the Netherlands; 4 GGzE Institute for Mental Health Care, Eindhoven, the Netherlands; 5 Arkin, Institute for Mental Health, Amsterdam, the Netherlands; a University Medical Center Amsterdam, Academic Medical Center, Department of Psychiatry, Amsterdam, the Netherlands; b Dimence Mental Health Care, Department of Psychosis, Deventer, the Netherlands

**Keywords:** Clinical staging, Cognitive performance, Course of disease, Schizophrenia spectrum disorders

## Abstract

**Background:**

Clinical staging has been developed to capture the large heterogeneity in schizophrenia spectrum disorders. Including cognitive performance in the staging model may improve its clinical validity. Moreover, cognitive functioning could predict transition across stages. However, current evidence of the association between cognition and clinical staging is inconsistent. Therefore, we aim to assess whether cognitive parameters are associated with clinical stages in a large sample of patients with schizophrenia spectrum disorders and to identify cognitive markers at baseline that are associated with stage-transition at three and six-year follow-up.

**Methods:**

We applied the staging model of Fusar-Poli et al. (2017) in 927 patients with non-affective psychotic disorders, assessed at baseline, and after three and six-year follow-up. Cognitive performance was assessed with a standard test battery. Generalized linear mixed models were used to analyze associations of cognitive performance with staging and stage-transition at follow-up.

**Results:**

Findings showed that higher stages of illness were significantly associated with lower processing speed (F = 3.688, *p* = 0.025) and deficits in working memory (F = 6.365, *p* = 0.002) across assessments. No associations between cognitive parameters at baseline and stage-transition at three- and six-year follow-up were found.

**Conclusion:**

We conclude that processing speed and working memory were modestly associated with higher stages of illness in schizophrenia spectrum disorders, thereby slightly improving its clinical validity. However, associations were small and we found no evidence for predictive validity.

## 1 Introduction

A relative new approach of refining the classification of schizophrenia spectrum disorders is clinical staging. This approach aims to map the heterogeneity in terms of symptom severity, level of remission and relapse. Thereby, creating a dynamic framework from early until late stages of disease, in which patients can change to severe or improved stages depending on their current psychiatric status (Mcgorry, 2007; McGorry et al., 2010). More recently, Fusar-Poli et al. (2017) and colleagues presented a revised staging model for early, secondary but also tertiary prevention (Fusar-Poli et al., 2017). In their review, the authors delineate diagnostics, targeted treatments selections and future challenges per clinical stage from clinical high risk for psychosis, first and recurrent psychosis to chronic illness. As the goal of treatment is to prevent transition to more advanced stages, this framework could also provide an opportunity for prompt recognition and intervention.

Cognitive deficits during childhood are among the earliest signs of distorted development resulting in psychosis (Kahn and Keefe, 2013), often followed by stabilization of cognitive performance around the occurrence of the first episode of psychosis (FEP) (Bora and Murray, 2014). However, after FEP, the relationship between severity of psychopathology and cognitive performance has been far from elucidated. Two prior meta-analyses from Ventura and colleagues revealed moderate associations between disorganization and negative symptoms with cognitive deficits in patients with schizophrenia(Ventura et al., 2010; Ventura et al., 2009). On the contrary, Rund et al. found in two studies that neither positive nor negative symptoms were associated with cognition in patients with schizophrenia. Yet, the same authors found that stable remission during the first year of psychosis was associated with improved cognitive performance (Rund et al., 2016). Moreover, an increased number of relapses within the first year of psychosis was also associated with lower scores on working memory and verbal learning (Rund et al., 2007). As the staging model is based on remission status, relapses and chronicity, the question emerges whether adding cognitive performance to the staging model might improve its clinical validity.

To the best of our knowledge, only three studies evaluated cognitive performance within the staging model. First, Godin et al. (2019) demonstrated that advanced disease stages were characterized by slower speed of processing and more severe executive disabilities. Nevertheless, post-hoc analyses concerning differences in cognition between specific stages were not performed(Godin et al., 2019). On the other hand, Tedja et al. (2017) found no significant differences on most cognitive subtests between stages in outpatients with schizophrenia spectrum disorders. In addition, the authors found that baseline cognitive parameters did not predict stage-transition at three-year follow-up(Tedja et al., 2017). Lastly, another cross-sectional study found significantly more decline in global cognitive functioning at more advanced disease stages in acutely admitted inpatients with schizophrenia spectrum disorders (Berendsen et al., 2021). The majority of the latter studies were cross-sectional or had a short-term follow-up, which is less suitable to evaluate stage-transition in relation to cognitive performance. Taken together, only limited and inconsistent evidence concerning associations between cognitive performance and clinical staging in schizophrenia spectrum disorders is currently available.

Therefore, we aim to assess whether cognitive parameters are associated with clinical stages in a large sample of patients with schizophrenia spectrum disorders and to identify cognitive markers at baseline that are associated with stage-transition at three- and six-year follow-up. We hypothesize that more advanced stages of illness will be associated with poorer cognitive performance, and that cognitive deficits at baseline will be associated with transition to more severe clinical stages at long-term follow-up.

## 2 Method

### 2.1 Study sample

The present study was conducted within the multicenter Genetic Risk and Outcome of Psychosis (GROUP) cohort study (Korver et al., 2012). Included patients were diagnosed with schizophrenia spectrum disorders and recruited from four university study-sites and their regional mental health care facilities in the Netherlands and Belgium. The total sample consisted of 1119 patients at baseline. After baseline measurement, patients were invited for two follow-up assessments after three and six years. Trained investigators conducted clinical interviews with patients and applied rating instruments.

### 2.2 Stage-assignment

We translated the recently proposed staging framework by Fusar-Poli et al. (2017) (Fusar-Poli et al., 2017) to our dataset using four variables: (1) definition of remission by Andreasen et al. (2005), measured with the Positive and Negative Syndrome Scale (PANSS) (Andreasen et al., 2005), (2) Global Assessment of Functioning (GAF), (3) cumulative number of episodes and (4) chronicity factor (Peralta and Cuesta, 1994; Aas, 2011; Fusar-Poli et al., 2017; Susser et al., 2000). The chronicity factor describes the severity and continuity of psychosis. We used score 5 and 6 from the chronicity factor, in which score 5 refers to a chronic illness, continuous psychosis with mild symptomatology and score 6 stands for a chronic illness, continuous psychosis with moderate or severe symptomatology. We divided stage 2 in three sub-stages, namely stage 2A defined as the first episode of psychosis (FEP) with symptomatic remission a GAF score >70, stage 2B FEP – incomplete remission and stage 2C FEP – currently psychotic with GAF score <70. Based on recent research findings (Berendsen et al., 2019), we subdivided stage 3B into stage 3B-1 (recurrent psychosis with more than two episodes and currently in symptomatic remission) and 3B-2 (multiple episodes and currently psychotic). For detailed information we refer to Table 1. For the multi cross-sectional analyses, we reduced the number of stages to ensure an adequate model fit. In stage 2 we included patients with a first episode of psychosis (stage 2A, 2B and 2C). In stage 3 we included patients with a single relapse of a psychotic disorder (stage 3A), multiple relapses in symptomatic remission and currently psychotic (stage 3B1 and 3B2), and stage 4 remained unchanged. This resulted in three stages.

**Table 1:** The adjusted Fusar-Poli staging model.

Staging model		Operationalization
Stage 2A	First episode of psychosis – currently in symptomatic remission	One psychotic episode
		Symptomatic remission + GAF >70
		Chronicity indicator scores <5
Stage 2B	First episode of psychosis – incomplete remission	One psychotic episode
		Symptomatic remission + GAF symptoms <70
		Chronicity indicator scores <5
Stage 2C	First episode of psychosis – currently psychotic	One psychotic episode
		Psychotic during measurement + GAF symptoms <70
		Chronicity indicator scores <5
Stage 3A	Single relapse of a psychotic disorder	Two psychotic episodes
		Psychotic during measurement or symptomatic remission
		Chronicity indicator scores <5
Stage 3B-1	Multiple relapses, symptomatic remission	>2 psychotic episodes
		Symptomatic remission
		Chronicity indicator scores <5
Stage 3B-2	Multiple relapses, currently psychotic	>2 psychotic episodes
		Psychotic during measurement
		Chronicity indicator scores <5
Stage 4	Chronic psychosis with severe persisting, unremitting illness	No remission
		Chronicity indicator scores 5 or 6: chronic illness with mild or mostly severe symptomatology

### 2.3 Definition of transition in stages at follow-up

To determine transition between stages, we used the original staging model, as described by Fusar-Poli in Table 1. We translated the model into three variables, declined, improved or stable. Decline towards more advanced stages of disease at 3 or 6-year follow-up compared to baseline was defined as follows: stage 2A towards 2B, or stage 2A, 2B, 2C towards any other higher stage (stage 3 or 4), stage 3A towards stage 3B-1, 3B-2 and 4, stage 3B-1 towards stage 3B-2 and 4, stage 3B-2 towards stage 4. Improvement in clinical stages at follow-up was defined as: stage 2B towards 2A, stage 2C towards 2A or 2B, stage 4 towards any other stage, stage 3B-2 towards 3B-1. Stable implies no change in stages at follow-up. We determined transition (improvement, stable or decline) in staging between baseline (T0) vs. three-year follow-up (T1) and baseline (T0) vs. six-year (T2) follow-up. Fig. 2, Fig. 3 provide a detailed graphical representation of stage-transition. To assess stage-transition it was necessary to have baseline staging data and at least one more assessment, otherwise patients would be excluded from the longitudinal analysis.

### 2.4 Cognitive measures

Neuropsychological assessments were conducted with a cognitive battery for domains similar to those defined in the MATRICS Consensus Cognitive Battery. Subtests of the WAIS-III were used to measure the domains of processing speed (digit-symbol coding task), working memory (arithmetic), reasoning and problem solving (block design task). A word learning task (the Auditory Verbal Learning Test) assessed verbal learning and memory, with outcome measure of immediate recall (15-word list, three learning trials) and retention rate (score on the delayed free recall trial divided by the maximum score on the learning trial after 20 min). The Continuous Performance Test was administered to test the domain of attention/vigilance, for which we used the average score of the overall accuracy score and mean reaction time(Firth et al., 2017). The tests were administered in a fixed order, testing time was approximately 90–120 min. Standardization of raw scores of individual cognitive tests was done by z-transformation. The z-transformation is calculated by subtracting the mean of all scores from each individual cognitive test score and subsequently dividing the remainder by the standard deviation of all scores. This z-transformation was done per cognitive domain per assessment, the resulting scores were used in the statistical analysis.

### 2.5 Covariates

We selected several confounders based on their a-priori association with cognitive decline or symptomatic outcome in schizophrenia spectrum disorders. We used the covariates age, gender (male or female), use of antipsychotic medication (yes or no) and educational level (primary, secondary or higher) in the primary analysis (Omachi and Sumiyoshi, 2018; Husa et al., 2017).

### 2.6 Statistical analysis

Differences between stages regarding baseline clinical and demographic characteristics were assessed by independent *t*-tests, analysis of variance or chi-square tests. To determine which cognitive domains were significantly associated with clinical stages across time, we performed generalized linear mixed models (GLMM) with staging as dependent variable and each cognitive domain as fixed effect, and a-priori selected covariates. Patients were included in the analyses if data were available for at least one time point (baseline, 3 years or 6 years) on the outcome variable of interest, because mixed modeling allowed to calculate valid estimates under the missing at random assumption, even if data for one or two time points were missing. Neither random intercept nor random slope were included, as they substantially increased Akaike Information Criterion (AIC) scores; this decision is supported by previous literature (Sommet and Morselli, 2017). Including all predetermined stages as outcome variable led to a low model fit, characterized by higher AIC scores and low percentage correctly predicted classifications. Therefore, we transformed the predetermined staging model into three stages, namely stage 2, 3 and 4 as described above. Fixed effects (cognitive domain and four covariates) were added *en bloc*. A lower AIC of the model after adding the covariates indicated a better model fit. Stage 2 (first episode of psychosis and incomplete remission of the first psychosis) was used as the reference category. In a subsequent step, we investigated whether baseline cognitive functioning scores were associated with change in staging at follow-up. We used the cognitive domains that were significantly associated with staging in the multi-crosssectional analysis. GLMM was conducted with baseline performance in cognitive domains added as fixed effects and stage-transition as outcome variable (with the ‘stable’ group as reference category). The same a-priori defined covariates were added to the model; we used no random effects or slopes, as they considerably increased AIC scores. In all analyses, *p*-values were calculated by the Kenward-Roger approach and Bonferroni-corrected post-hoc analyses were conducted if a significant fixed effect was found. Statistical analyses were conducted with Statistical Package for the Social Sciences (SPSS) version 26. Data release 7.0 was used for the analyses.

## 3 Results

### 3.1 Study sample characteristics

Detailed clinical and demographic information is shown in Table 2. We found significant differences between stages in age (F (dF = 5, *N* = 927) = 11.334, *p* < 0.001) and educational level (X^2^ (dF = 10, *N* = 925) = 42.200, *p* < 0.001). Reason for exclusion was one or more missing variables necessary for stage-assignment. At baseline, we included 927 patients, at three-year follow-up 661 patients and six-year follow-up 547 patients. Patients that could not be assigned to any of the stages did not differ significantly from the remainder of the cohort in terms of duration of illness or the investigated cognitive domains. They were, however, characterized by significantly younger age (*T* = 2.361, *p* = 0.018), higher GAF symptomatology scores (*T* = 3.223, *p* = 0.001) and lower number of episodes (*T* = 0.015, *p* = 0.001).

**Table 2:** Baseline clinical and demographic characteristics.

	Stage 2A (*N* = 91)	Stage 2B (*N* = 132)	Stage 2C (*N* = 213)	Stage 3A (*N* = 218)	Stage 3B-1 (*N* = 83)	Stage 3B-2 (*N* = 62)	Stage 4 (*N* = 128)	Between groups	dF	*p*-Value
Age (SD)	26.8 (7.4)	25.8 (6.3)	25.0 (6.4)	28.5 (7.3)	30.0 (8.6)	27.7 (7.0)	29.7 (7.7)	11.334	6	<0.001
Gender % female	34.1%	21.2%	17.2%	19.3%	32.5%	19.4%	20.3%	17.258	6	0.008
Antipsychotic medication										
% Using	78.0%	91.7%	93.0%	87.6%	91.6%	90.3%	92.2%	41.220	18	0.001
% Not using	0.0%	0.0%	0.5%	1.4%	0.0%	0.0%	0.0%			
% Unknown	1.1%	2.3%	4.7%	4.1%	0.0%	1.6%	4.7%			
% Missing	20.9%%	6.1%	1.9%	6.9%	8.4%	8.1%	3.1%			
Educational level								51.226	12	<0.001
% Primary	4.4%	10.6%	16.4%	10.6%	7.2%	12.9%	25.0%			
% Secondary	49.5%	50.0%	48.4%	43.1%	43.4%	61.3%	54.7%			
% Higher	46.2%	39.4%	35.2%	46.3%	49.4%	25.8%	20.3%			

### 3.2 Transition in stages at follow-up

We had sufficient data to determine the stage of illness of 595 patients at three-year follow-up and of 497 patients at six-year follow-up. At three-year follow-up 40.7% (*N* = 242) of the patients remained stable in terms of stages, 15.8% improved (*N* = 94) and 43.5% (*N* = 259) declined in staging in comparison to baseline. At six-year follow-up in total 32.0% (*N* = 159) remained stable, 15.5% (*N* = 77) improved and 52.5% (*N* = 261) declined in staging.

### 3.3 Association of cognitive performance with clinical stages

**Fig. 1:**
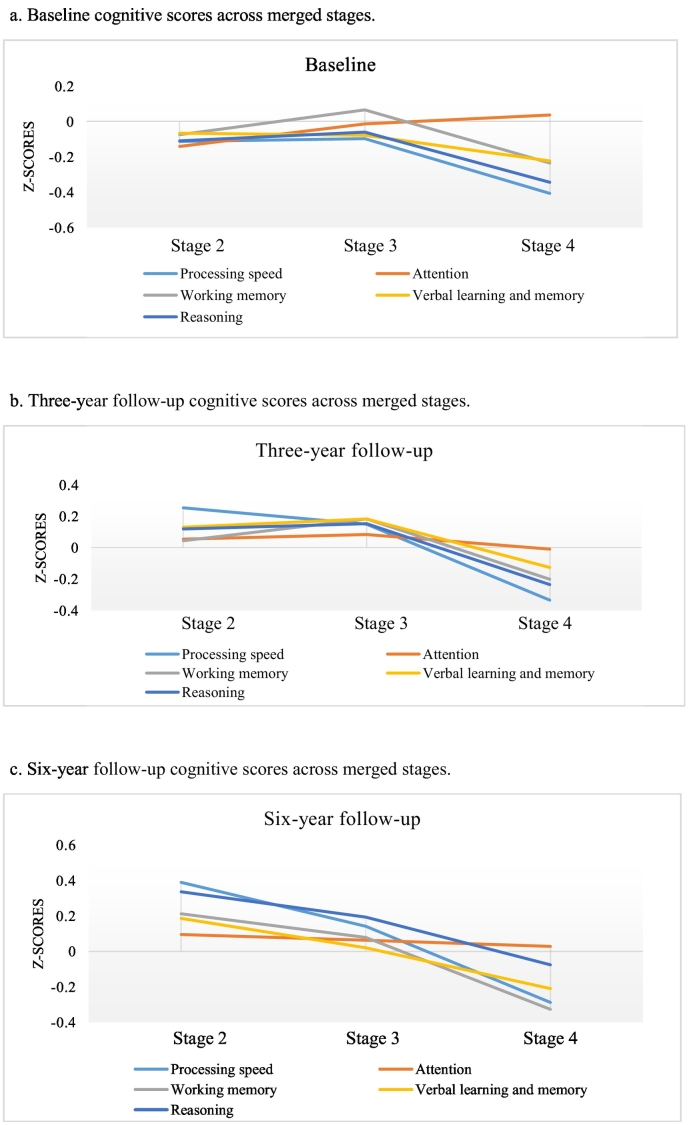
Cognitive scores across merged stages. a. Baseline cognitive scores across merged stages. b. Three-year follow-up cognitive scores across merged stages. c. Six-year follow-up cognitive scores across merged stages.

Cognitive scores across the merged stages are shown in Fig. 1a–c, with detailed information found in the supplement Table 2. In addition, the original stages in relation to cognitive performance are shown in the supplement Table 1 and Fig. 3a–c. Results of the association between cognitive domains and staging across time are shown in Table 3. Fixed effects show significant associations for processing speed (F = 3.688, *p* = 0.025) and working memory (F = 6.365, *p* = 0.002) with clinical staging, other cognitive domains were not significantly associated with staging. Table 3 also shows that covariates antipsychotic medication, age and educational level were significantly associated with stages in all models (*p* < 0.001). Pairwise comparisons with stage 2 as the reference category are shown in Table 4. Results indicate that only stage 4 showed significantly poorer performance in the processing speed (estimate: −0.160, *p* = 0.035) and working memory task (estimate = −0.201, *p* = 0.009), after controlling for covariates. Table 5 demonstrates that neither baseline processing speed nor working memory were significantly associated with stage-transition at three- and six-year follow-up.

**Table 3:** Fixed effects of generalized linear mixed models (P-value <0.05) regarding the multi cross-sectional associations between clinical stages and individual cognitive subtests.

Clinical staging	F-value	P-Value
Corrected model	13.256	**<0.001**
Processing speed	3.688	**0.025**
Educational level	8.636	**<0.001**
Age	63.296	**<0.001**
Antipsychotic medication	4.428	**<0.001**
Gender	1.458	0.233
Corrected model	13.162	**<0.001**
Attention	0.978	0.376
Educational level	13.512	**<0.001**
Age	64.464	**<0.001**
Antipsychotic medication	4.682	**<0.001**
Gender	2.644	0.071
Corrected model	13.567	**<0.001**
Working memory	6.365	**0.002**
Educational level	6.927	**<0.001**
Age	66.210	**<0.001**
Antipsychotic medication	4.627	**<0.001**
Gender	3.519	**0.030**
Corrected model	13.177	**<0.001**
Verbal learning and memory	0.689	0.502
Educational level	11.428	**<0.001**
Age	66.214	**<0.001**
Antipsychotic medication	4.862	**<0.001**
Gender	2.035	0.131
Corrected model	13.315	**<0.001**
Reasoning	2.967	0.052
Educational level	9.463	**<0.001**
Age	66.382	**<0.001**
Antipsychotic medication	4.919	**<0.001**
Gender	2.606	0.074

**Table 4:** Post-hoc tests of between group differences of generalized linear mixed models (P-Value < 0.05) regarding associations between clinical stages and cognitive performance (stage 2 reference category).

		Estimate	Standard error	*P*-Value
Stage 3	Intercept	−2.365	0.3146	**<0.001**
	Processing speed	0.032	0.0583	0.583
	Educational level (primary)	−0.147	0.2108	0.486
	Educational level (secondary)	−0.216	0.1151	0.060
	Age	0.081	0.0079	**<0.001**
	Antipsychotic medication – not using	0.094	0.3716	0.801
	Antipsychotic medication –using	0.518	0.1523	**0.001**
	Gender	−0.010	0.1273	0.939
Stage 4	Intercept	−5.020	0.4486	**<0.001**
	Processing speed	−0.160	0.0759	**0.035**
	Educational level (primary)	0.984	0.2445	**<0.001**
	Educational level (secondary)	0.491	0.1570	**0.002**
	Age	0.095	0.0097	**<0.001**
	Antipsychotic medication not using	0.941	0.5177	0.069
	Antipsychotic medication - using	1.154	0.2464	**<0.001**
	Gender	0.259	0.1746	0.139
Stage 3	Intercept	−2.336	0.3022	**<0.001**
	Working memory	0.056	0.0589	0.346
	Educational level (primary)	−0.139	0.2139	0.515
	Educational level (secondary)	−0.204	0.1202	0.090
	Age	0.080	0.0079	**<0.001**
	Antipsychotic medication – not using	0.177	0.3697	0.633
	Antipsychotic medication – using	0.527	0.1514	**0.001**
	Gender	−0.043	0.1285	0.738
Stage 4	Intercept	−5.255	0.4367	**<0.001**
	Working memory	−0.201	0.0765	**0.009**
	Educational level (primary)	0.928	0.2489	**<0.001**
	Educational level (secondary)	0.423	0.1633	**0.010**
	Age	0.099	0.0096	**<0.001**
	Antipsychotic medication – not using	0.911	0.5217	0.081
	Antipsychotic - using	1.185	0.2460	**<0.001**
	Gender	0.386	0.1761	**0.028**

**Table 5:** Fixed effects of generalized linear mixed models regarding the associations between stage-transition at three and six-year follow-up with baseline cognitive performance.

Stage-transition	F-value	*p*-Value
Corrected model	0.746	0.706
Processing speed	2.444	0.087
Working memory	0.280	0.756
Educational level	0.841	0.499
Age	3.104	0.045
Antipsychotic medication	0.000	1.000
Gender	0.050	0.951

**Fig. 2:**
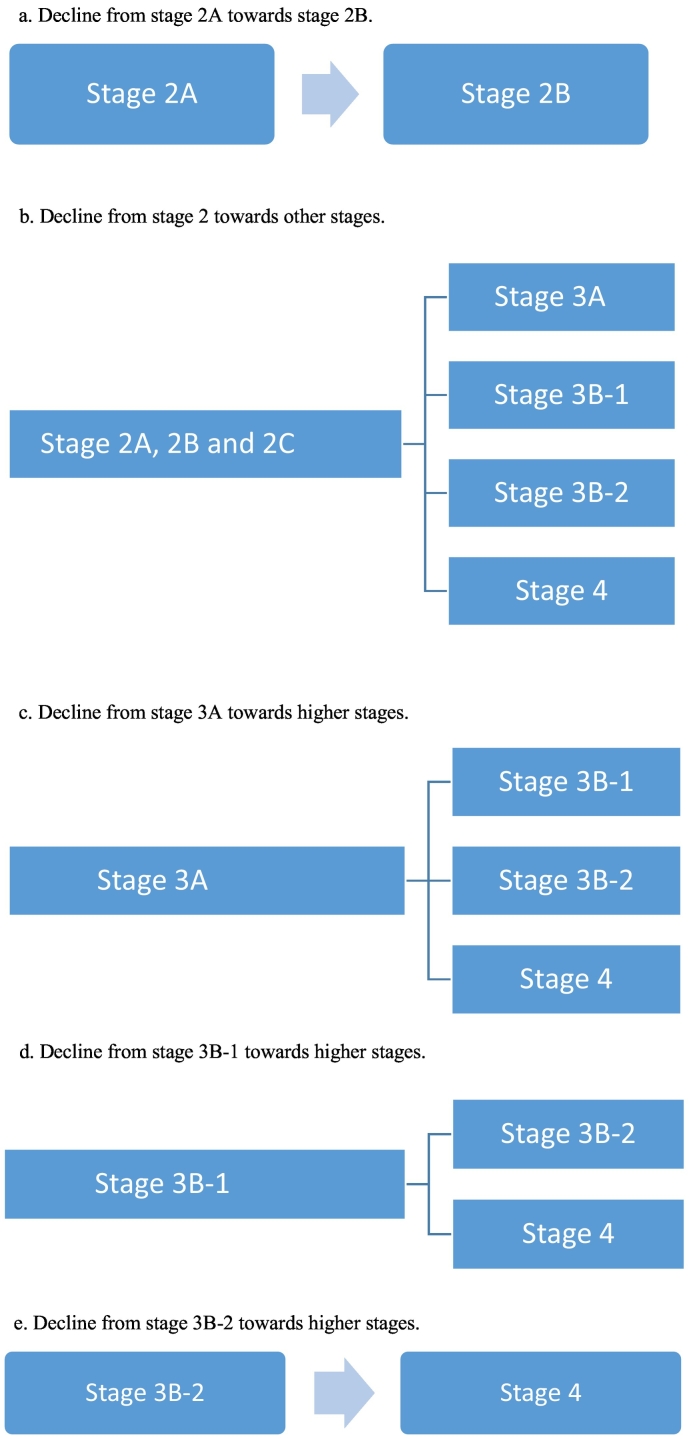
Illustration of decline in stage-transition. a. Decline from stage 2A towards stage 2B. b. Decline from stage 2A, B, C towards other stages. c. Decline from stage 3A towards higher stages. d. Decline from stage 3B-1 towards higher stages. e. Decline from stage 3B-2 towards higher stages.

**Fig. 3:**
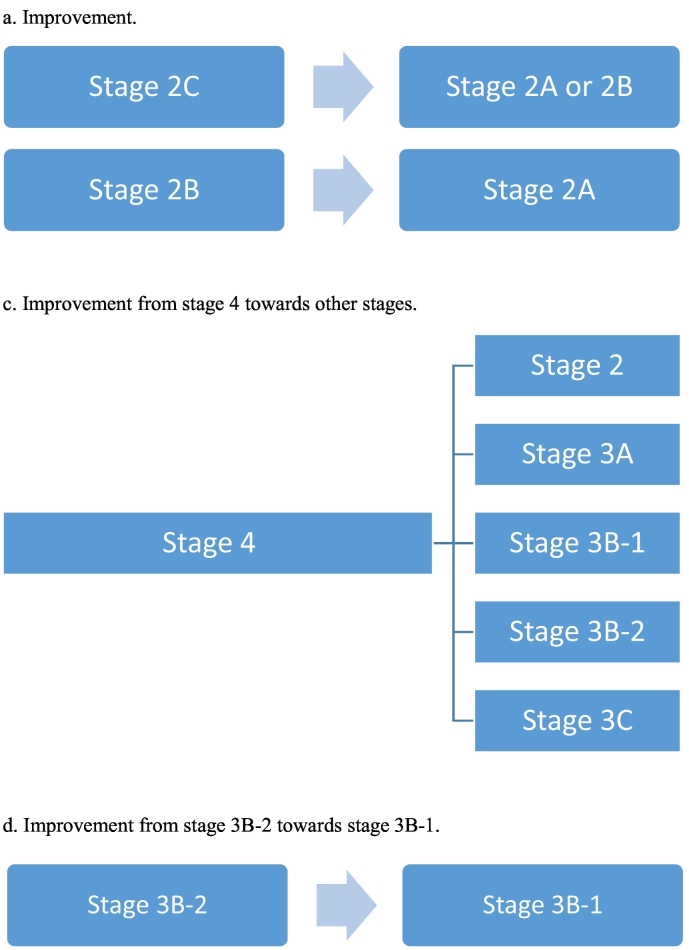
Illustration of improvement in stage-transition. a. Improvementt from stage 2B towards stage 2A, stage 2C towards 2A or stage 2B. b. Improvement from stage 4 towards other stages. c. Improvement from stage 3B-2 towards stage 3B-1.

## 4 Discussion

The present study aimed to explore cognitive performance in relation to the clinical staging model of schizophrenia spectrum disorders. Consistent with our hypothesis, we found significant multi cross-sectional associations between lower cognitive performance, specifically speed of processing and working memory, with the most advanced stage of illness. Suggesting that specific subgroups of patients with schizophrenia spectrum disorders with chronic symptoms experience more cognitive deficits. Thus, complementing the staging model with working memory and processing speed could slightly improve its clinical validity. However, these cognitive parameters were not associated with stage-transition at three and six-year follow-up.

So far only few studies investigated the associations between cognitive functioning and stages (Berendsen et al., 2021; Godin et al., 2019; Tedja et al., 2017). Two studies also showed associations of cognitive deficits with higher stages of illness. While research on a smaller subsample of GROUP participants, performed by Tedja et al. (2017), only reported that the WAIS - information subtest was significantly different between stages at baseline, not corrected for educational level. Differential results can be partly explained by relatively low sample size, less follow-up assessments and stricter inclusion criteria.

A larger body of evidence focused on the relationship between cognition and aspects of psychopathology, such as symptom severity, relapse or chronicity. Their results showed that more relapses were associated with deficits in working memory, while speed of information processing was not associated with severity of psychopathology or relapses (Rund et al., 2007; Rund et al., 2016). From a wider perspective, a meta-analysis by Forbes et al. demonstrated that working memory deficits are more present in patients with multiple-episode versus first-episode schizophrenia (Forbes et al., 2009). Consistent with our findings, a meta-analysis by Dickinson et al. (2007) demonstrated that processing speed is at least weakly associated with severity of clinical symptoms in patients with schizophrenia (Dickinson et al., 2007). The authors even proposed that processing speed may be a robust assessment tool for patients with schizophrenia, considering its relation to long-term functional outcome.

However, we found no evidence for predictive validity of cognitive performance in the staging model. Consequently, the found associations elucidate only a modest proportion of the complex interaction of prognostic factors that determine outcome in patients with schizophrenia spectrum disorders. Other factors such as support of relatives, substance abuse or treatment compliance may be more strongly related to the chronicity and relapse of psychotic symptoms (de Haan et al., 2007; Weiden et al., 2004; Alvarez-Jimenez et al., 2012). This also suggests that the status of psychopathology assessed by clinical staging is partly unrelated to the cognitive performance in patients with schizophrenia spectrum disorders. It may therefore well be that unrelated mechanisms could cause the expression of cognitive deficits and co-occurrence of severity or recurrence of psychotic symptoms. The presumption of the staging model is that higher stages are accompanied by progressive cognitive impairment. However, it could well be that improvement in staging is also characterized by improved or stable cognitive functioning, this question remains open for further investigation. Overall, our results emphasized the relative importance of working memory and processing speed for the advanced stages of illness. Cognitive performance is clinically important and treatment interventions are needed. In fact, several interventions could aid in increasing cognitive performance in patients with schizophrenia spectrum disorders. For instance, cognitive remediation therapy (CRT) or aerobic exercise training are both proven effective interventions to improve cognitive performance (Firth et al., 2017; Vita et al., 2021). Importantly, in CRT lower baseline severity of symptoms was associated with greater improvement in global cognition after treatment, underlining the value of symptomatic remission for treatment of cognitive performance in schizophrenia.

The main strength of this study is that we are the first to evaluate associations of cognitive performance with clinical stages in patients with schizophrenia spectrum disorders at long-term follow-up, among a large cohort of patients. Importantly, we evaluated whether cognitive markers predicted long-term stage transition. However, our study should be viewed in light of two limitations. Firstly, patients included in the GROUP study represent a relatively high functioning proportion of patients, limiting the generalizability of results. Secondly, we were not able to include the original staging model as proposed by Fusar-Poli et al. (2017) in the generalized linear mixed model. We were obliged to reduce the number of stages to ensure an adequate model fit. Inevitably, this approach precludes more detailed findings. Thirdly, we specified stage 2 and stage 3A with GAF scores and severity of psychopathology, while we did not use the GAF score for other stages. As a result, relatively high functioning patients (GAF scores >70) could be classified in the higher stages. It is well known that general functioning is a predictor of cognitive performance and not including the GAF in these stages could therefore have influenced our findings(Santesteban-Echarri et al., 2017). However, this would have led to a substantial loss of sample size and we therefore chose to exclude the GAF in classifying these stages. In conclusion, associations between stages of illness with working memory and speed of processing were robust but minor. Including cognitive parameters into the staging model may slightly improve its validity. At the same time, we may also conclude that other important predictive factors may cause transition across stages, and distinct pathways could cause psychotic symptoms and cognitive deficits. Future research may therefore focus on the mechanisms related to differential outcome in cognitive deficits and psychopathology. Moreover, further research is needed to elucidate which factors, other than cognitive performance, contribute to understanding transition across stages over time.

## Ethic approval

The study was approved by the Medical Ethics Committee of the Academic Medical Center of Utrecht. All patients gave written informed consent before enrollment in the study.

## Funding

The infrastructure for the GROUP study is funded through the Geestkracht programme of the Dutch Health Research Council (Zon-Mw, grant number 10-000-1001), and matching funds from participating pharmaceutical companies (10.13039/501100013327Lundbeck, 10.13039/100004325AstraZeneca, 10.13039/100004312Eli Lilly, 10.13039/100014554Janssen Cilag) and universities and mental health care organizations (Amsterdam: Academic Psychiatric Centre of the 10.13039/501100003180Academic Medical Center and the mental health institutions: 10.13039/501100003292GGZ Ingeest, Arkin, Dijk en Duin, GGZ Rivierduinen, 10.13039/501100003061Erasmus Medical Centre, GGZ Noord Holland Noord. Groningen: 10.13039/501100005075University Medical Center Groningen and the mental health institutions: 10.13039/501100003294Lentis, 10.13039/501100003295GGZ Friesland, 10.13039/501100003296GGZ Drenthe, Dimence, Mediant, GGNet Warnsveld, Yulius Dordrecht and Parnassia psycho-medical center The Hague. Maastricht: 10.13039/501100004528Maastricht University Medical Centre and the mental health institutions: GGzE, GGZ Breburg, GGZ Oost-Brabant, Vincent van Gogh voor Geestelijke Gezondheid, Mondriaan, Virenze riagg, Zuyderland GGZ, MET ggz, Universitair Centrum Sint-Jozef Kortenberg, 10.13039/501100007660CAPRI University of Antwerp, PC Ziekeren Sint-Truiden, PZ Sancta Maria Sint-Truiden, GGZ Overpelt, OPZ Rekem. Utrecht: 10.13039/501100003761University Medical Center Utrecht and the mental health institutions Altrecht, GGZ Centraal and 10.13039/100002465Delta).

## CRediT authorship contribution statement

SB, EN, NFS, LDH, MJT contributed to the study design, methodology, statistical analysis and writing of the manuscript. SB and MJT performed the statistical analysis, SB wrote the first version of the manuscript and MJT and LDH provided study supervision. The GROUP authors performed data-collection and provided critical comments to the manuscript. All authors contributed to and have approved the final manuscript.

## Declaration of competing interest

None.

## Appendix A Supplementary data

Supplementary material Image 1
