# Supplementary material for: Association of cognitive performance with clinical staging in schizophrenia spectrum disorders: a prospective 6-year follow-up study
Source: Schizophr Res Cogn. 2021 Dec 14;28:100232. doi: 10.1016/j.scog.2021.100232 (PMC8866148; doi:10.1016/j.scog.2021.100232)
Supplement: Supplementary file 1 — Supplementary material [file mmc1.docx]

**Table 1. Cognitive performance across stages (z-scores)**

|  |  | Mean (SD) | Stage 2A (N=91) | Stage 2B (N=132) | Stage 2C  (N=213) | Stage 3A (N=218) | Stage 3B-1 (N=83) | Stage 3B-2 (N=62) | Stage 4 (N=128) |
| --- | --- | --- | --- | --- | --- | --- | --- | --- | --- |
| Baseline | Processing speed (SD) | -0.121 (0.98) | 0.151 (0.97) | 0.009 (0.96) | -0.294 (0.920) | -0.077 (0.97) | -0.012 (1.03) | -0.258 (0.82) | -0.406 (0.95) |
|  | Attention (SD) | -0.072 (0.79) | -0.175 (0.79) | 0.020 (0.69) | -0.219 (0.72) | -0.006 (0.65) | 0.112 (0.45) | -0.217 (1.37) | 0.038 (0.55) |
|  | Working memory (SD) | -0.042 (0.99) | 0.103 (1.06)) | 0.110 (0.92) | 0.110 (0.92) | 0.022 (0.97) | 0.206 (0.91) | 0.053 (1.03) | -0.234 (1.01) |
|  | Verbal learning and memory (SD) | -0.097 (0.78) | 0.085 (0.73) | -0.051 (0.76) | -0.135 (0.82) | -0.046 (0.79) | -0.068 (0.75) | -0.186 (0.84) | -0.222 (0.80) |
|  | Reasoning (SD) | -0.0840 (0.97) | -0.177 (1.00) | 0.0590 (0.99) | -0.216 (1.02) | -0.060 (0.99) | 0.120 (0.96) | -0.263 (0.97) | -0.343 (0.98) |
|  |  |  |  |  |  |  |  |  |  |
| Three-year follow-up | Processing speed (SD) | 0.085 (1.00) | 0.480 (1.06) | 0.259 (0.96) | -0.741 (0.79) | 0.293 (0.96) | 0.226 (0.96) | -0.376 (0.82) | -0.334 (0.97) |
|  | Attention (SD) | 0.047 (0.60) | 0.067 (0.51) | 0.063 (0.53) | 0.044 (0.37) | 0.052 (0.63) | 0.180 (0.51) | 0.006 (0.79) | -0.009 (0.73) |
|  | Working memory (SD) | 0.076 (1.07) | 0.035 (1.08) | 0.062 (0.97) | -0.168 (1.12) | 0.236 (0.97) | 0.270 (0.95) | -0.114 (0.95) | -0.201 (0.94) |
|  | Verbal learning and memory (SD) | 0.110 (0.79) | 0.208 (0.78) | 0.185 (0.77) | -0.416 (0.79) | 0.186 (0.78) | 0.328 (0.74) | -0.079 (0.60) | -0.126 (0.88) |
|  | Reasoning (SD) | 0.079 (0.94) | 0.145 (0.91) | 0.160 (0.89) | -0.165 (1.01) | 0.180 (0.91) | 0.258 (0.97) | -0.103 (0.89) | -0.235 (1.03) |
|  |  |  |  |  |  |  |  |  |  |
| Six-year follow-up | Processing speed (SD) | 0.104 (1.02) | 0.677 (1.00) | 0.322 (1.06) | -0.103 (1.18) | 0.304 (0.94) | 0.142 (0.99) | -0.194 (1.00) | -0.288 (0.99) |
|  | Attention (SD) | 0.059 (0.61) | 0.035 (0.46) | 0.155 (0.50) | 0.111 (0.50) | 0.029 (0.65) | 0.082 (0.42) | 0.095 (0.74) | 0.028 (0.69) |
|  | Working memory (SD) | -0.004 (1.03) | 0.321 (1.02) | 0.190 (1.21) | -0.030 (0.86) | 0.166 (0.95) | 0.081 (1.03) | -0.107 (1.03) | -0.327 (0.97) |
|  | Verbal learning and memory (SD) | 0.032 (0.82) | 0.339 (0.71) | 0.015 (0.82) | 0.039 (0.75) | 0.147 (0.81) | 0.012 (0.82) | -0.225 (0.82) | -0.210 (0.82) |
|  | Reasoning (SD) | 0.147 (1.04) | 0.371 (0.96) | 0.448 (0.99) | -0.051 (1.26) | 0.212 (1.00) | 0.211 (1.05) | 0.121 (1.09) | -0.076 (1.07) |

**Table 2. Cognitive performance across merged stages (z-scores)**

|  |  | Stage 2 (N=436) | Stage 3 (N=363) | Stage 4 (N=128) |
| --- | --- | --- | --- | --- |
| Baseline | Processing speed (SD) | -0.112 (0.96) | -0.096 (0.96) | -0.406 (0.95) |
|  | Attention (SD) | -0.140 (0.73) | -0.012 (0.80) | 0.038 (0.55) |
|  | Working memory (SD) | -0.073 (0.99) | 0.067 (0.97) | -0.234 (1.01) |
|  | Verbal learning and memory (SD) | -0.065 (0.79) | -0.077 (0.79) | -0.222 (0.80) |
|  | Reasoning (SD) | -0.108 (1.00) | -0.059 (0.98) | -0.343 (0.98) |
|  |  |  |  |  |
| Three-year follow-up | Processing speed (SD) | 0.254 (1.06) | 0.149 (0.96) | -0.334 (0.97) |
|  | Attention (SD) | 0.055 (0.50) | 0.084 (0.63) | -0.009 (0.73) |
|  | Working memory (SD) | 0.044 (1.04) | 0.183 (0.97) | -0.201 (0.94) |
|  | Verbal learning and memory (SD) | 0.131 (0.81) | 0.183 (0.75) | -0.126 (0.88) |
|  | Reasoning (SD) | 0.119 (0.92) | 0.153 (0.93) | -0.235 (1.03) |
|  |  |  |  |  |
| Six-year follow-up | Processing speed (SD) | 0.389 (1.07) | 0.141 (0.98) | -0.288 (0.99) |
|  | Attention (SD) | 0.095 (0.47) | 0.063 (0.59) | 0.028 (0.69) |
|  | Working memory (SD) | 0.212 (1.07) | 0.078 (1.00) | -0.327 (0.97) |
|  | Verbal learning and memory (SD) | 0.186 (0.75) | 0.020 (0.82) | -0.210 (0.82) |
|  | Reasoning (SD) | 0.336 (0.98) | 0.193 (1.03) | -0.076 (1.07) |

Figure 3. Cognitive scores of original stages in relation to cognitive performance.

Figure 3a. Baseline cognitive scores with original stages.

Figure 3b. Three-year follow-up cognitive scores with original stages.

Figure 3c. Six-year follow-up cognitive scores with original stages.
